# Radiotherapy-drug combinations in the treatment of glioblastoma: a brief review

**DOI:** 10.2217/cns-2021-0015

**Published:** 2022-05-23

**Authors:** Patrick G McAleavey, Gerard M Walls, Anthony J Chalmers

**Affiliations:** 1 School of Medicine, Dentistry & Biomedical Sciences, Queen's University Belfast, 97 Lisburn Road, Belfast, BT9 7BL, N. Ireland; 2 Cancer Centre Belfast City Hospital, Lisburn Road, Belfast, BT9 7AB, N. Ireland; 3 Patrick G Johnston Centre for Cancer Research, Jubilee Road, Belfast, BT9 7AE, N. Ireland; 4 Institute of Cancer Sciences, University of Glasgow, Garscube Estate, Switchback Road, Bearsden, G61 1QH, Scotland

**Keywords:** chemotherapy, glioblastoma, radiosensitizers, radiotherapy, trial design

## Abstract

Glioblastoma (GBM) accounts for over 50% of gliomas and carries the worst prognosis of all solid tumors. Owing to the limited local control afforded by surgery alone, efficacious adjuvant treatments such as radiotherapy (RT) and chemotherapy are fundamental in achieving durable disease control. The best clinical outcomes are achieved with tri-modality treatment consisting of surgery, RT and systemic therapy. While RT-chemotherapy combination regimens are well established in oncology, this approach was largely unsuccessful in GBM until the introduction of temozolomide. The success of this combination has stimulated the search for other candidate drugs for concomitant use with RT in GBM. This review seeks to collate the current evidence for these agents and synthesize possible future directions for the field.

Glioblastoma (GBM) is the most common primary brain cancer in adults with an age-standardized incidence of 4.64 per 100,000 in England. GBM carries the worst prognosis of all solid tumors, with a median overall survival (OS) of approximately 12 months [1]. Furthermore, patients with GBM often experience extremely poor quality of life, largely due to tumor related symptoms and treatment side effects [2].

Optimal treatment of GBM involves a multi-modality approach. Gross total resection of GBM is not curative [3], but maximal extent of resection is associated with increased survival [4,5]. Factors such as poor performance status at baseline, deep-seated location, extent of infiltration and location within an eloquent site each impact on the achievable degree of debulking.

The incorporation of molecular tumor analysis into routine clinical practice has transformed treatment decisions for the GBM cohort. In particular, the degree of methylation of the promoter region of the DNA repair gene *MGMT* has proven utility as a prognostic and predictive biomarker. Methylation of the MGMT promoter region silences this gene, attenuating repair of the DNA damage generated by the alkylating chemotherapy agent temozolomide (TMZ) and hence enhancing sensitivity to this compound [6].

Other significant genetic abnormalities commonly reported on in reflex testing panels for glioma include mutations of IDH, TERT and components of the PI3K/AKT signaling pathway including EGFR, EGFRvIII and PTEN. Of note, high grade gliomas with 1p19q co-deletion and/or IDH mutation are no longer categorized as GBM, even if they exhibit the conventional histopathological features [7]. Profiling of the genetic landscape of glioma has certainly improved classification, grading and prognostication, and informed treatment decisions to some extent, but most patients with GBM still receive generic treatment [8].

Operative techniques have also evolved in the last decade with the implementation of pre-operative and intra-operative neuro-navigation, achieved with the use of MRI and diffusion-tensor imaging (DTI) [9]. Tumors can also be better visualized intra-operatively to achieve more extensive resection with the use of 5-aminolevulinic acid (5-ALA)-induced fluorescence. This technique enables the neurosurgeon to more easily identify and resect tumor while preserving more normal brain parenchyma [10]. Another neurosurgical technique that may increase the extent of ‘safe resection’ is awake craniotomy. The goal of this technique is to identify and preserve important functional cortical areas intra-operatively, thereby reducing the risk of significant post-operative neurological deficit [11].

Following maximal feasible resection, patients usually proceed to a course of radical radiotherapy, consisting of daily outpatient treatment [12]. Younger patients with good performance status (WHO PS 0-1) are scheduled to receive 6 weeks of daily treatments (total dose 60 Gray in 30 fractions), whereas older patients (65–70 years or older) or those with a poorer performance status (WHO PS 2) will undergo 3 weeks of treatment (total dose 40 Gray in 15 fractions). Targeting of residual tumor and/or resection cavity has become more precise due to the fusion of MRI and CT images for radiotherapy planning, and modern treatment planning algorithms and linear accelerators facilitate more accurate, intensity-modulated radiotherapy (IMRT) [13]. Taken together, these computing and engineering advances have resulted in more uniform dosing of the tumor and reduced doses to adjacent normal brain tissues and other critical structures.

Despite the implementation of these developments, GBM remains a devastating disease, being classified as a ‘cancer of unmet need’ by Cancer Research UK [14], and thus novel treatment paradigms are urgently sought. In our view, improving the effectiveness of post-operative treatment is currently the most viable approach, and real-world data showing benefit from adding temozolomide to radiotherapy provides evidence that novel RT-drug combinations have potential to improve outcomes [15].

While many potential agents are still in the early stages of development, preclinical data suggest that synergistic effects are achievable and several novel combinations are either undergoing early phase evaluation or are due to be investigated in upcoming human studies. The purpose of this review is to convene the recent progress in the field of RT-drug combinations in GBM and identify future avenues for research.

## Temozolomide

Temozolomide (TMZ) is an oral agent which is hydrolyzed to 3-methyl-(triazen-1-yl)imidazole-4-carboxamide (MTIC) having crossed the blood–brain barrier. MTIC has a preponderance for alkylating/methylating guanine bases at the N-7 or O-6 positions, and such damage may lead to cell death if not resolved by DNA repair mechanisms, of which MGMT is the most effective [16].

**Figure 1.**
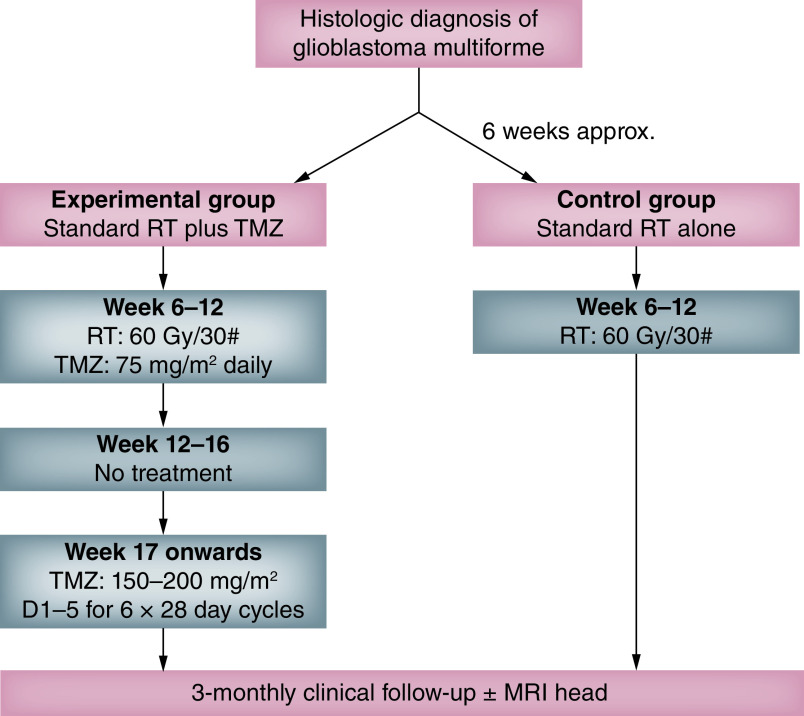
Adapted from trial design of the 2005 Stupp study. D: Day; RT: Radiotherapy; TMZ: Temozolomide.

TMZ has been included in the standard of care of GBM since the landmark trial published by Stupp *et al.* in 2005. This study investigated patients 18–70 years of age with newly diagnosed GBM and a WHO performance status of 0–2. Patients were randomized to receive standard RT (60 Gy in 30 fractions over 6 weeks) with or without concomitant daily TMZ (75 mg/m^2^) followed by adjuvant TMZ (200 mg/m^2^, 5 days every 4 weeks) for 6 months (Figure 1). Patients receiving TMZ plus RT had a 37% lower relative risk of death, with a median survival benefit of 2.5 months compared with RT alone. The 2-year survival rate was 26.5% in the group receiving TMZ plus RT, and 10.4% in the group receiving RT only [17].

*Post hoc* analysis of tumor tissue from the Stupp trial found that patients whose tumors exhibited methylation of the MGMT promoter region experienced improved survival compared with patients whose tumors lacked MGMT methylation, and derived significant additional survival benefit from the addition of TMZ. While the survival benefit associated with TMZ in patients whose tumors lacked MGMT methylation was not statistically significant, in the absence of any other effective systemic agents, concomitant RT-TMZ has become the ‘gold standard’ regimen for all GBM irrespective of methylation status [6].

The findings of the Stupp trial did not apply to patients aged over 70, however. Given the peak age of onset of GBM is in the seventh decade, and the increasingly ageing populations in many countries, a separate study in this important patient subpopulation was carried out. Building on previous work showing equivalent (or superior) outcomes when shorter, hypofractionated radiotherapy schedules were used in elderly patients [18,19], Perry *et al.* prospectively investigated a cohort of 562 patients with newly diagnosed GBM aged between 65 and 90 (median 73 years) and asked whether addition of concomitant and adjuvant TMZ to short course RT (40 Gy in 15#) would improve outcomes. This study demonstrated a statistically significant improvement in OS in the group receiving TMZ (9.3 v. 7.6 months), and confirmed the predilection for better outcomes when tumors exhibited MGMT promoter methylation [20].

## Combination of RT-TMZ with other agents

Since the superiority of RT-TMZ over radiotherapy alone was established, multiple additional agents have been investigated for their utility alongside this combination. One example, the AVAglio trial, investigated the addition of bevacizumab for newly diagnosed GBM. This placebo-controlled Phase III trial assessed the addition of 2-weekly bevacizumab 10 mg/kg to the previously outlined RT-TMZ combination. No OS benefit was found for the addition of bevacizumab, and the rate of grade ≥3 adverse events was higher than in the control group (32.5 vs 15.8%, p < 0.05) [21]. Similar findings were published simultaneously by a separate group investigating the same combination, where bevacizumab in combination with standard of care TMZ did not increase survival as first-line post-operative treatment [22]. In elderly patients who may not be able to tolerate standard of care postoperative RT/TMZ regimens, monotherapy with either hypofractionated RT or TMZ may be pursued. The ARTE trial investigated the addition of bevacizumab to such patients receiving RT monotherapy [23]. Although this initial study did not identify a survival benefit with the addition of bevacizumab, a post-hoc ancillary imaging study found that patients with larger tumors, defined as tumors with preoperative contrast enhancement greater than or equal to 3.1 cm^3^, benefitted from the addition of bevacizumab to RT monotherapy [24].

A Phase II study investigated the addition of celecoxib, isotretinoin and/or thalidomide to dose-dense adjuvant TMZ following chemoradiation [25]. This Phase II study included eight treatment arms, each one receiving a regimen of RT and dose-dense TMZ plus either one of the additional investigative agents, combinations of two agents, or all three. Although not combining these agents with RT directly, this innovative trial demonstrated the feasibility of ‘doublet’, ‘triplet’ and ‘quadruplet’ therapy in radically treated GBM. No statistically significant benefit was seen in OS or progression-free survival for the combinations groups adjuvantly, though sequencing these drugs concurrently with radiation has not been tested.

Other cytotoxic agents have shown preclinical efficacy when combined with RT in animal studies. Intra-arterial carboplatin has been used as a lone salvage therapy in recurrent GBM [26], but has also demonstrated preclinical synergistic effects when used in combination with RT [27]. A current Phase I/II trial (NCT03672721) is investigating the use of intra-arterial carboplatin in combination with RT in the setting of relapsed GBM. Other cytotoxic drugs such as motexafin gadolinium and farnesyltransferase inhibitors did not produce any clinical benefit when combined with RT [28,29].

## PARP inhibitors

PARP is a nuclear protein involved in base excision repair as part of the DNA damage response (DDR) provoked by RT and alkylating agents such as TMZ. It has been found to be overexpressed in GBM and other high-grade glial tumors, which may explain some of the radioresistance exhibited by these tumors [30,31]. PARP inhibition is predicted to increase the therapeutic ratio of RT because rapidly proliferating tumor cells are selectively sensitized to radiation, without such effects on non-proliferating cells in the surrounding normal brain parenchyma [32].

The OPARATIC trial (NCT03212742) investigated olaparib and TMZ concomitantly (without RT) in a Phase I study in recurrent GBM. This trial established the maximum tolerated dose (MTD) of olaparib as 150 mg once daily, 3 days per week, when given alongside daily low dose TMZ as would be delivered in combination with standard IMRT (60 Gy in 30 fractions). The primary aim of this study was to determine the recommended Phase II dose of olaparib, though efficacy signals were also generated for the combination as well as confirmation of the safety profile. Translational results of the Phase I trial confirmed the capacity of olaparib to penetrate the blood–brain barrier at radiosensitizing concentrations [33].

Of note, agents in this class of drugs have led to exaggerated hematological toxicity in combination trials with TMZ, prompting TMZ dose reductions and/or intermittent dosing of PARP inhibitors [34]. As some evidence supports the acceptability of omitting TMZ in GBM with unmethylated MGMT status [35], the ongoing PARADIGM-2 trial (ISRCTN51253312) will go some way to further exploring research questions relating to the combination of olaparib, RT and TMZ in GBM. This program consists of two parallel Phase I studies, assessing olaparib-RT-TMZ and olaparib-RT in patients with newly diagnosed GBM with methylated and unmethylated MGMT status, respectively [36].

The randomized Phase II VERTU trial (ACTRN12615000407594) investigated the use of veliparib with concurrent RT (60 Gy in 30 fractions) in unmethylated GBM, followed by six cycles of adjuvant TMZ in combination with veliparib [37]. This trial demonstrated that these veliparib combinations were well tolerated but provided no clinical benefit in the population of patients with MGMT unmethylated GBM [38].

Inactivation of the mismatch repair (MMR) gene *MSH6*, and to a lesser extent *MSH2, MLH1* and *PMS2*, has been implicated in acquired TMZ resistance in both IDH mutant and IDH wild-type recurrent GBM [39]. PARP inhibitors have been shown to restore TMZ sensitivity in *MSH6*-deficient *in vivo* GBM models with veliparib, where potent suppression of tumor growth was observed when combined with TMZ [40]. This additional potential indication for PARP inhibition requires further exploration.

## EGFR inhibitors

In GBM the EGFR is frequently found to be amplified, mutated, or overexpressed [41]. The most frequently expressed EGFR variant in GBM, EGFRvIII (seen in 30% of patients [42]) comprises a constitutively active yet impaired tyrosine kinase receptor that is ligand-independent, activating anti-apoptotic and pro-invasive signaling pathways [43]. While it has been suggested that co-expression of the oncogene EGFRvIII with the PTEN mutation is associated with response to pharmacological EGFR inhibition in GBM *in vitro* models [41], at present there is no robust evidence to suggest that this response is transferrable to clinical practice. EGFR parameters are regularly included when profiling the molecular background of GBM samples and although aberrant EGFR is common and actionable in extracranial primary malignancies, very few patients with GBM respond to EGFR tyrosine kinase inhibitors (TKIs).

In terms of clinical studies, only Phase II evidence exists to date. A 2009 study indicated that erlotinib, a first-generation EGFR inhibitor, had efficacy in combination with RT-TMZ (NCT00187486) [44]. A median OS of 19.3 months was achieved in this modest cohort of 65 patients, compared with 14.1 months in a historical control group. However a small study (n = 27) investigating erlotinib plus RT-TMZ at similar dose-levels reported a median OS of only 8.6 months (NCT00274833). This trial was closed early due to excessive rates of treatment-related deaths (n = 3) which included pneumocystis jirovecii pneumonia (PJP) (n = 1) and refractory bone marrow aplasia (n = 2). Treatment failure signals were also higher than expected, with 81% patients coming off-study due to progressive disease [45]. Further studies investigating erlotinib have failed to produce positive results, and subset analyses have not found a specific molecular profile that benefits from the addition of this drug [46,47].

Osimertinib is a third generation oral TKI known for its utility in targeting EGFR with T790M mutations in non-small cell lung cancer (NSCLC) [48]. Unlike earlier EGFR TKIs, osimertinib has favorable blood–brain barrier penetration meaning intracranial control in EGFR-mutant NSCLC has improved dramatically [49]. Preclinical data also pointed to possible efficacy of osimertinib in EGFRvIII-positive GBM. *In vitro* investigations demonstrated that osimertinib has high affinity for the EGFRvIII tyrosine kinase, blocking downstream signaling in pathways involved with cell proliferation. *In vivo* investigations demonstrated an OS benefit in immunodeficient mice receiving a clinically relevant dose of osimertinib when the mice were implanted intracranially with GBM stem cells [50].

A case report of a young patient with multifocal GBM who received off-label osimertinib for recurrent disease demonstrated complete response in one of her lesions which expressed EGFR C628F and A289V mutations. However there was a mixed response overall, with a separate lesion with EGFRvIII positivity progressing, suggesting that hitherto unknown molecular mechanisms interact with those currently embedded in practice [51]. These data exemplify the heterogenous nature of GBM and the challenges involved in its treatment. Ongoing clinical trials seek to define the role of osimertinib in patients with recurrent GBM with EGFR amplification [52].

## Wee1 inhibitors

Wee1 kinase is a critical downstream mediator of the p53-independent G2 checkpoint which is activated by tumor cells in response to RT-induced DNA damage. Since the vast majority of GBM exhibit defects in G1/S checkpoint function, the tumor cells are thought to be more dependent on the G2/M checkpoint. Wee1 is activated by several DDR kinases, and in turn increases levels of inactivated CDK1 leading to G2 checkpoint activation which provides additional time for repair of DNA damage before cells attempt to undergo mitosis. Wee1 is therefore an attractive target involved in p53-independent G2 checkpoint activation, and several agents are undergoing development for use in GBM [53].

Adavosertib, a potent inhibitor of Wee1 kinase, produced a dose-dependent attenuation of G2 checkpoint arrest after RT-induced damage in GBM cell lines, and increased mitotic catastrophe [54]. Pharmacokinetic analysis of adavosertib in a Phase I clinical trial reported good tumor penetration in patients with first relapse of GBM, with pharmacologically relevant concentrations being reached [55]. This contrasted with prior murine model results where low concentrations of Wee1 inhibitor were observed in both brain and tumor tissue, using orthotopic GBM xenografts [56]. A dose-finding study (NCT01849146) is currently underway for AZD1775 in combination with RT-TMZ for GBM [57].

## ATM & ATR inhibitors

The repertoire of DNA damage caused by ionizing radiation includes single-strand breaks (SSBs) and more lethally, double-strand breaks (DSBs). The latter activate the ATM and ATR kinases, which then activate downstream checkpoint kinases Chk1 and Chk2. This process results in cell cycle arrest, allowing time for repair of the DSBs by either homologous recombination (HR) or non-homologous end joining (NHEJ) machinery. ATM also plays a role in promoting DSB repair. Inhibition of ATM or ATR therefore increases cell death in response to radiation, and the potency of these effects indicates considerable potential as radiosensitizers in tumors where radiotherapy outcomes remain poor such as GBM [58].

There is compelling preliminary preclinical evidence describing the potent radiosensitizing effects of ATM inhibitors such as KU-60019, which attenuated radioresistance in a panel of GBM cell lines [59,60]. The ATM-mediated DSB repair pathway in GBM cancer stem cells has also been shown to be abrogated by the ATM inhibitor KU-55933, leading to profound radiosensitization with enhancement ratios of 2.6–3.5, depending on the human GBM cell line tested [61].

The third-generation ATM inhibitor AZD1390 potently inhibits ATM *in vitro*, modulating the DDR and and acting as a radiosensitizer with particularly marked effects in p53-deficient GBM cell lines [62]. Having been designed specifically to achieve brain penetrance, this agent is currently being trialed in combination with radiation a ‘first in human’ study in UK and USA (NCT03423628). This innovative study consists of three treatment arms recruiting patients with newly diagnosed (60 Gy in 30 fractions) or relapsed GBM (35 Gy in 10 fractions), or brain metastases (30 Gy in 10 fractions; whole or partial-brain). The estimated study completion date is July 2022 [63]. At the time of writing, no clinical studies have been published in this area.

ATR inhibitors are less potent modulators of the DSB-induced DDR pathway, however show promise when combined with DNA damaging agents including radiation [64]. Preclinical models have shown that inhibition of ATR and the associated G2-M pathway along with PARP inhibition achieves optimal radiosensitization through parallel inhibition of DDR pathways [61]. At present, there are no active ATR inhibitor studies in GBM patients, although the PATRIOT (NCT02223923) trial is investigating the ATR inhibitor AZD6738 both as monotherapy and as part of combination therapy with palliative RT in the setting of solid tumors [65], and a Phase I dose escalation study of a new ATR-targeted agent elimusertib in combination with radiation in relapsed and newly diagnosed GBM is in advanced development.

## Immune checkpoint inhibitors

Compared with other primary tumors, such as metastatic melanoma and NSCLC, GBM has a lower mutational burden, and is locally immunosuppressive. The resulting tumor microenvironment is detrimental for immunotherapy such as immune checkpoint inhibitors, which act in part through local T-cell interactions. The aforementioned relative impermeability of the BBB may also be a factor limiting the efficacy of immunotherapy in GBM. Local immunosuppression in GBM is associated with recruitment of regulatory T-cells which attenuate anti-tumor immune responses, as well as the actions of pro-tumorigenic factor-secreting macrophages. GBM associated monocytes have also been shown to express high levels of the programmed death-1 ligand, leading to increased death of T cells. These are some of the hurdles that immunotherapy faces in the context of GBM [66,67].

Nivolumab is a human immunoglobulin (Ig) G4 monoclonal antibody which inhibits the programed death-1 (PD-1) checkpoint receptor and has shown activity in a number of extracranial tumor types. A Phase III trial (NCT02017717) compared nivolumab to bevacizumab in the setting of first-recurrence GBM, after initial standard of care surgery, TMZ and RT [68]. Phase II results were not recapitulated however [69], and the trial failed to demonstrate improved median OS compared with bevacizumab by pre-specified criteria [70]. Of note, the control arm, bevaciuzumab, is not an internationally recognized therapy in this setting. Checkmate-143 also had several embedded Phase I arms that enabled preliminary testing of combined anti-CTLA4/anti-PD1 blockade with ipilimumab/nivolumab, and further clinical evaluation is underway [70]. The Phase III Checkmate-548 trial (NCT02667587), which investigated the addition of nivolumab to standard of care in the setting of MGMT-methylated relpased GBM, also failed to meet its primary end point of increased survival [71].

## Conclusion

The last two decades have been an exciting era for GBM researchers, heralded by the adoption of TMZ into standard-of-care and the emergence of novel therapeutic agents such as PARP, ATM and ATR inhibitors, with promising early data. There remains a critical need to test these agents in an efficient and informative manner, and to develop entirely new strategies for the treatment of this difficult disease. The heterogenous and challenging biology of GBM means that multi-modality therapy is likely to provide the best opportunity to improve outcomes, and hence the evaluation of combination therapies comprising surgery, RT and systemic agents in efficient contemporaneously developed trial designs should be prioritized.

## Future perspective

While clinical outcomes appear unlikely to improve substantially in the short-term, the emerging treatment options (Table 1) and the current sustained research investment are combining to make the current era an exciting one for clinicians treating patients with GBM. If this investment is sustained by health policymakers, and technology developed in other primary tumor sites proves to be transferrable to GBM, improvements in survival and quality of life can be envisaged in the medium-term.

**Table 1.** Summary of key drug-RT combination studies in glioblastoma.

Trial number	Investigated regime	Phase	Radiotherapy	Posology	Number of patients	Result
NCT00006353^†^	TMZ + RT	III	60 Gy/30#	75 mg/m^2^/day TMZ max 49 days	575	Positive
NCT00943826^†^	Bevacizumab + TMZ + RT	III	60 Gy/30#	Bevacizumab 10 mg/kg IV q2w TMZ standard of care	921	Negative
NCT03672721^†^	Carboplatin + RT	I/II	15–35 Gy/10#	400 mg/m^2^ intra-arterial carboplatin before 1st and 6th fraction, then monthly	Approx. 35	Awaited
ISRCTN51253312^‡^	Olaparib + RT ± TMZ	I	60 Gy/30#	Parallel 1 (MGMT-methylated cohort) – intermittent daily olaparib defined by dose cohort + standard of care Parallel 2 (MGMT-unmethylated) – continuous daily olaparib	Approx. 50	Awaited
NCT01849146^†^	Adavosertib (AZD1775) + TMZ ± RT	I	Arm I: 60 Gy/30# Arm II: None	Arm I – AZD1775 PO on days 1, 3 and 5 or 1–5 weekly + standard of care TMZ Arm II – AZD1775 PO QD on days 1, 3 and 5 or 1–5 weekly, TMZ PO QD on days 1–5	114	Awaited
NCT03423628^†^	AZD1390 + TMZ + RT	I	Arm I: 35 Gy/10# Arm II: 30 Gy/10# Arm III: 60 Gy/30#	AZD1390 administered in 3 cycles depending on arm: Cycle 0 (arms A and C): 1 dose prior to radiation therapy Cycle 1 (all arms): intermittent or continuous dosing during radiation therapy (except for first 2 cohorts of arm A) Cycle 2 (arms A and C): 2 weeks adjuvant treatment after radiation therapy	132	Awaited
NCT02667587^†^	Nivolumab + RT + TMZ	III	60 Gy/30#	Nivolumab 3 mg/kg IV every 2 weeks TMZ standard of care	693	Negative

† Available at https://clinicaltrials.gov/

‡ Available at https://www.isrctn.com/ISRCTN51253312

IV: Intravenous; PO: Oral; q2w: Every 2 weeks; QD: Four times daily; RT: Radiotherapy; TMZ: Temozolomide.

The utility of biological therapies targeting ‘checkpoints’ in the immune system, one branch of immunotherapy, has increased exponentially across multiple tumor sites [72]. Combining immune checkpoint inhibitors with RT is of particular interest in GBM, based on the potential for RT to increase tumor cell antigen presentation and immune stimulation [73]. However as outlined, results to date have been disappointing. Nonetheless, the programed death ligand-1 (PD-L1) inhibitor durvalumab is currently under investigation in recurrent GBM in combination with stereotactic ablative radiotherapy (SABR) (NCT02866747). This Phase I/II trial investigates SABR (24 Gy in 3 fractions) with or without adjuvant durvalumab (1500 mg) with a primary end point of OS [74]. The first durvalumab infusion will take place on the day of the final fraction of SABR and will be administered every 4 weeks until relapse or toxicity.

Other ongoing trials are investigating immune checkpoint inhibitors in combination with additional therapies such as interstitial laser thermotherapy and molecular targeted agents [67], however these are all in early stages. The need to identify patients who are more likely to respond to checkpoint inhibition is currently a key gap where novel biomarkers are being sought. It has been shown in other cancers that higher levels of PD-L1, microsatellite instability (MSI), and higher tumor mutation burden (TMB) together predict for a positive response to this type of immunotherapy [75]. This composite biomarker has very low prevalence in GBM, however, so selecting patients for trials and treatments remains a challenge.

Immunotherapy as a treatment paradigm also carries specific risks in patients with GBM. Exaggerated immune responses within the cranial vault, such as immunotherapy-related pseudoprogression, cytokine storm and autoimmune encephalitis could provoke acute deterioration requiring hospitalization [76]. Some patients with GBM may have greater difficulty managing and reporting some of the severe toxicities of immunotherapy in comparison with other patient cohorts due to the neurocognitive complications of prior surgery or chemoradiotherapy [77,78].

Other forms of immunotherapy include chimeric antigen receptor T-cell (CAR-T) therapy and viral therapy. CAR-T therapy is most established in the treatment of hematological cancers [79] and involves the generation and administration of modified T cells with an extracellular domain that recognizes tumor-specific antigens and an intracellular domain that promotes T-cell activation. The modified cells can initiate an immune response against tumor cells that possess the specific antigen recognized by the extracellular domain. In GBM, relevant antigens that could be targeted include IL-13Ra2, EGFRvIII and HER2 [75]. A case study of a patient with multifocal GBM receiving CAR-T against IL-13Ra2 demonstrated regression of all lesions for a period of 7.5 months [80]. Promising results have also been documented for CAR-T with specificity for the other antigens listed [81]. Interestingly, preclinical data have demonstrated synergistic effects of CAR-T and subtherapeutic doses of RT in orthotopic mouse GBM models [82]. These mice were also protected against tumor rechallenge in the contralateral cerebral hemisphere. Further clinical work is required but there is reason to believe that this form of immunotherapy may confer clinical benefit to a subgroup of GBM patients in the future.

Viral therapy involves infecting tumor cells with a specific virus that may have cytolytic effects that activate the innate immune system, promote cytokine release and potentially create a long-term immune response. The Toca-5 trial had reported promising results in Phase I and II trials investigating viral therapy but unfortunately the Phase III trial was recently terminated by its sponsors having failed to meet the primary end point of increased OS [83]. The oncolytic virus HSV-1 G207 has also been investigated in GBM. Preclinical studies of this modified HSV showed that its effect was enhanced by clinically relevant doses of RT and increased survival in orthotopic models. Promising effects were observed clinically after stereotactic inoculation of the oncolytic virus into human GBM at a median of 18 months after diagnosis: radiological improvements were seen on MRI and some patients benefitted from a further median survival of 7.5 months after inoculation (n = 9) [84]. This small study illustrates the potential for this oncolytic virus to improve outcomes in GBM and further investigation is warranted.

Vaccine therapy is another form of immunotherapy currently being tested in GBM patients after recent success in other solid tumors. First results from a Phase III clinical trial of an autologous tumor lysate-pulsed dendritic cell vaccine (DCVax^®^-L) showed that the addition of this vaccine therapy to standard of care treatment is safe in these patients. Furthermore, a post-operative median OS of 23.1 months was reported in the intent-to-treat population [85]. However the median OS assessment in this trial should be viewed with caution: since the vaccine therapy had been shown to provide survival benefits based on MGMT status previously, a cohort of patients in the control cohort of this trial received the dendritic cell vaccine after reaching the trial primary end-point, therefore rendering the OS assessment uncontrolled and unreliable [86].

The ACT IV trial (NCT01480479) investigated rindopepimut in combination with TMZ in the EGFRvIII-expressing population of GBM patients. Rindopepimut is a vaccine composed of an EGFRvIII-specific peptide conjugated to keyhole limpet hemocyanin. It was administered alongside TMZ via monthly intradermal injection in postoperative patients who had completed standard chemoradiotherapy. The control group, rather than receiving placebo, received keyhole limpet hemocyanin to adjust for local adverse effects seen with rindopepimut, such as erythema, pruritus and rash. Analysis of this combination showed no survival benefit in the experiment group, and the trial was ceased early due to futility [87].

Personalized neoantigen vaccines have also been investigated in GBM. Neoantigens are derived from tumor-specific protein-coding mutations, and vaccination with these can generate robust immune responses. One small Phase Ib study showed that in patients with newly diagnosed GBM that did not receive dexamethasone, postoperative personalized multi-epitope neovaccine administration resulted in corresponding T-cell tumor infiltration found on examination of tissue taken when subsequent debulking occurred [88]. Cancer vaccines such as this have the propensity to stimulate an intratumoral immune response [89], however the clinical significance of this neoantigen vaccine in GBM is yet to be demonstrated.

Various other drug-RT regimens are currently being investigated, including 2-OHOA, a sphingomyelin synthase-1 activator, in the CLINGLIO trial (NCT04250922). This Phase II/III study involves this first-in-class therapy in combination with current standard of care in patients with newly diagnosed GBM. Animal studies and other preclinical data have suggested 2-OHOA to have a greater anti-tumor effect than TMZ with a higher efficacy and lower tumor relapse rate [90,91].

The Phase III MIRAGE trial (NCT03345095) investigated the use of the proteasome inhibitor marizomib in combination with current standard of care in patients with newly diagnosed GBM [92]. Marizomib is a naturally occurring marine compound which binds to and inhibits a protein complex involved in the degradation of defunct proteins. This trial followed from previous successes with this drug in Phase I and II studies [93], and included a total of 749 patients, of which the experimental cohort received concomitant and adjuvant marizomib as well as TMZ. The experimental arm did not benefit from increased OS or PFS and there was a significantly higher rate of treatment-related adverse events, particularly neurologic and psychiatric, in this group. Further analyses of this trial are ongoing [94].

One of the biggest obstacles to development of new drug-RT combinations for GBM, as well as other treatment modalities, is tumor heterogeneity. Ultimately there is an unmet need for the expansion of molecular profiling as well as discovery of new biomarkers to guide and develop a strategy of personalized medicine in GBM. Emerging data indicate that optimal outcomes are likely to be achieved when multimodality treatments are used, guided by biomarkers.

With respect to novel biomarkers, there has recently been interest in the use of radiomics for prognostication in GBM [95]. Radiomics refers to the extraction and high throughput analysis of large amounts of quantitative imaging data. Two forms of radiomics are in use: ‘hand-crafted’ and ‘deep learning’; the latter allows for increased automaticity and a higher yield of outcome discriminators [96]. In GBM, radiomics offers potential to explore genetic and molecular characteristics of patients' tumors in a non-invasive fashion. Specialized software can extract thousands of imaging features from CT and MRI data and evidence-based patterns may be identified that are associated with clinical and molecular characteristics. The utility of radiomics in tumor classification will potentially assist in treatment (combination) selection and prediction of treatment response. Research to date shows that radiomic tool performance is highest when integrated with classic clinical parameters [95].

As the development of novel anticancer treatments accelerates and the burden of trial regulation and cost rises, novel, efficient study designs are of increasing interest. Multi-arm, multi-stage platform studies have been successfully instigated in other cancer settings [97,98] and carry the benefit of widening the investigative scope of a research group, with embedded translational research, along with more efficient use of research funding and resources. These trials are an emerging technology in themselves, and demand high levels of statistical input. One such trial in GBM, INSIGhT, is a platform trial investigating novel biomarkers for newly diagnosed GBM [99].

The GBM AGILE trial (NCT03970447) is a novel multi-arm clinical trial design that is utilizing ‘adaptive randomization’ in an effort to detect activity signals at an earlier stage than in conventional trials. A principle behind the design is that if one drug is recognized to be outperforming another, more patients will be enrolled into that treatment arm allowing the investigative power to be increased. In theory this will streamline the process and allow earlier recognition of effective treatments. GBM AGILE aims to enroll 1030 patients and is investigating drugs include maintenance paxalisib (an mTOR/PI3K inhibitor), maintenance regorafenib (a multitargeted TKI), and maintenance VAL-083 (an alkylating agent). These drugs are being investigated in both the setting of newly diagnosed, MGMT unmethylated GBM, newly diagnosed, MGMT methylated GBM (VAL-083 only), and recurrent GBM. The trial completion date is estimated to be June 2024 [100].

**Executive summary**

- The heterogenous and aggressive nature of glioblastoma (GBM) accounts for poor prognosis despite tri-modality treatment strategies and extensive research.
- The introduction of temozolomide into the standard of care regimen provided significant clinical benefits in multiple patient cohorts.
- Molecular profiling is increasingly used in GBM and provides opportunities for the induction of personalized medicine.
- PARP overexpression in GBM accounts for a degree of radioresistance, and inhibition of this molecule therefore has the potential to radiosensitize tumor, maximizing the benefit achieved from radiotherapy. One such inhibitory drug, olaparib, is being investigated in the PARADIGM-2 trial.
- Aberrant EGFR expression is actionable in extracranial malignancies and common in GBM; however, at present, there is no robust clinical evidence to support its use for radical intent.
- Wee1 is an important mediator of the response to radiotherapy-induced DNA damage, and inhibition of this molecule represents another potential therapeutic avenue. ATM and ATR inhibition also act to limit the cellular repair mechanisms post-radiotherapy in GBM cells, and trials are ongoing in this area.
- GBM is a locally immunosuppressive tumor, limiting the benefits that can be afforded by immunotherapy.
- As well as seeking to identify agents that potentiate radiotherapy, recent interest in the development of novel trial design strives to improve outcomes for patients with GBM.
